# Physiologically based pharmacokinetic modeling of daptomycin dose optimization in pediatric patients with renal impairment

**DOI:** 10.3389/fphar.2022.838599

**Published:** 2022-08-16

**Authors:** Lingling Ye, Xiang You, Jie Zhou, Chaohui Wu, Meng Ke, Wanhong Wu, Pinfang Huang, Cuihong Lin

**Affiliations:** Department of Pharmacy, The First Affiliated Hospital of Fujian Medical University, Fuzhou, China

**Keywords:** daptomycin, physiologically based pharmacokinetic model, pediatric patients with renal impairment, pharmacokinetics, pharmacodynamics

## Abstract

**Background and Objective:** Daptomycin is used to treat Gram-positive infections in adults and children and its dosing varies among different age groups. We focused on the pharmacokinetics of daptomycin in children with renal impairment, which has not been evaluated.

**Methods:** A physiologically based pharmacokinetic (PBPK) model of daptomycin was established and validated to simulate its disposition in healthy populations and adults with renal impairment, along with a daptomycin exposure simulated in pediatric patients with renal impairment.

**Results:** The simulated PBPK modeling results for various regimens of intravenously administered daptomycin were consistent with observed data according to the fold error below the threshold of 2. The C_max_ and AUC of daptomycin did not differ significantly between children with mild-to-moderate renal impairment and healthy children. The AUC increased by an average of 1.55-fold and 1.85-fold in severe renal impairment and end-stage renal disease, respectively. The changes were more significant in younger children and could reach a more than 2-fold change. This scenario necessitates further daptomycin dose adjustments.

**Conclusion:** Dose adjustments take into account the efficacy and safety of the drug; however, the steady-state C_min_ of daptomycin may be above 24.3 mg/L in a few instances. We recommend monitoring creatine phosphokinase more than once a week when using daptomycin in children with renal impairment.

## Introduction

Daptomycin (CUBICIN^®^) belongs to a new class of cyclic lipopeptide antibiotics (Heidary et al., 2018). It is approved for the treatment of complicated skin and skin structure infections (cSSSI), *Staphylococcus aureus* bloodstream infections (*S. aureus* bacteremia) in adults and pediatric patients 1–17 years of age, and right-sided infective endocarditis in adults (FDA, 2020).

Although the use of vancomycin is associated with a high failure rate in the treatment of methicillin-resistant *S. aureus* (MRSA) blood infections (Kalil et al., 2014; van et al., 2012; Shariati et al., 2020), it is still the mainstream clinical treatment method. In contrast, daptomycin treatment is associated with lower clinical failure and 30-days mortality rates than vancomycin treatment as reported in MRSA blood-infection studies (Claeys et al., 2016). In addition, daptomycin is an effective option for treating vancomycin-resistant enterococci (Melese et al., 2020; Shi et al., 2020). Multiple guidelines also recommend daptomycin for the treatment of *S. aureus* bacteremia or cSSSI (Stevens et al., 2014; Habib et al., 2015; Baddour et al., 2015; Sartelli et al., 2018).

The recommended daptomycin dosing regimens in adults are 4 mg/kg q24 h (once every 24 h) for the treatment of cSSSI and 6 mg/kg q24 h for the treatment of *S. aureus* bacteremia by infusion over 30 min or injection over 2 min. The dosing interval should be prolonged to q48 h (once every 48 h) in adult patients when the creatinine clearance rate (CL_CR_) is <30 ml/min. However, the dosing regimens for pediatric patients are more complex because daptomycin clearance is higher in younger than in older children (Principi et al., 2015). Hence, daptomycin dosing varies among different age groups. However, daptomycin dosage adjustments for pediatric patients with renal impairment have not been established (FDA, 2020).

The pharmacokinetics (PK) of daptomycin is generally linear, and a steady state is reached after 3 days of intravenous, once-daily administration (Hair and Keam, 2007). Daptomycin is primarily bound to serum albumin with a concentration-independent protein binding rate of 90%–93%. The volume of distribution at steady-state (V_ss_) of daptomycin in healthy adult subjects is dose-independent and approximately 0.1 L/kg. The mean plasma half-life of daptomycin is approximately 8–9 h. It tends to be prolonged by deteriorated renal function, for even up to 28 h, because renal excretion is the primary elimination route. Approximately 5.7% of the dose is found in feces, but according to *in vitro* studies, daptomycin is not metabolized by human liver microsomes (FDA, 2020). Therefore, significant drug-drug interactions between daptomycin and drugs metabolized by these systems are not expected. In addition, the antibiotic can be injected within approximately 2 min, making it a simple and safe choice for adult outpatients.

Based on PK studies of daptomycin in adult subjects with various degrees of renal function (Yabuno et al., 2013; Chaves et al., 2014; Kullar et al., 2014; Xu et al., 2017; Xie et al., 2020), dose adjustments are recommended in adult patients whose CL_CR_ is less than 30 ml/min, whereas no dosage adjustment is necessary for adults with mild-to-moderate renal impairment. However, there are no data for pediatric patients with renal impairment. To complement the lack of research in this area, our research team used physiologically based pharmacokinetic software to combine the drug parameters of daptomycin with the physiological parameters of children with renal impairment. Based on several studies conducted by our research team and others on PBPK modeling for successfully predicting drug concentrations and drug-drug and drug-disease interactions in special populations (Zhang et al., 2019; Chen et al., 2020; Ye et al., 2020; You et al., 2020; Li et al., 2021; Liu et al., 2021), we established a new PBPK model of daptomycin for simulating the daptomycin PK in pediatric patients with renal impairment and evaluating the drug’s pharmacodynamics (PD).

## Materials and methods

### Description of PBPK model development workflow

**FIGURE 1:**
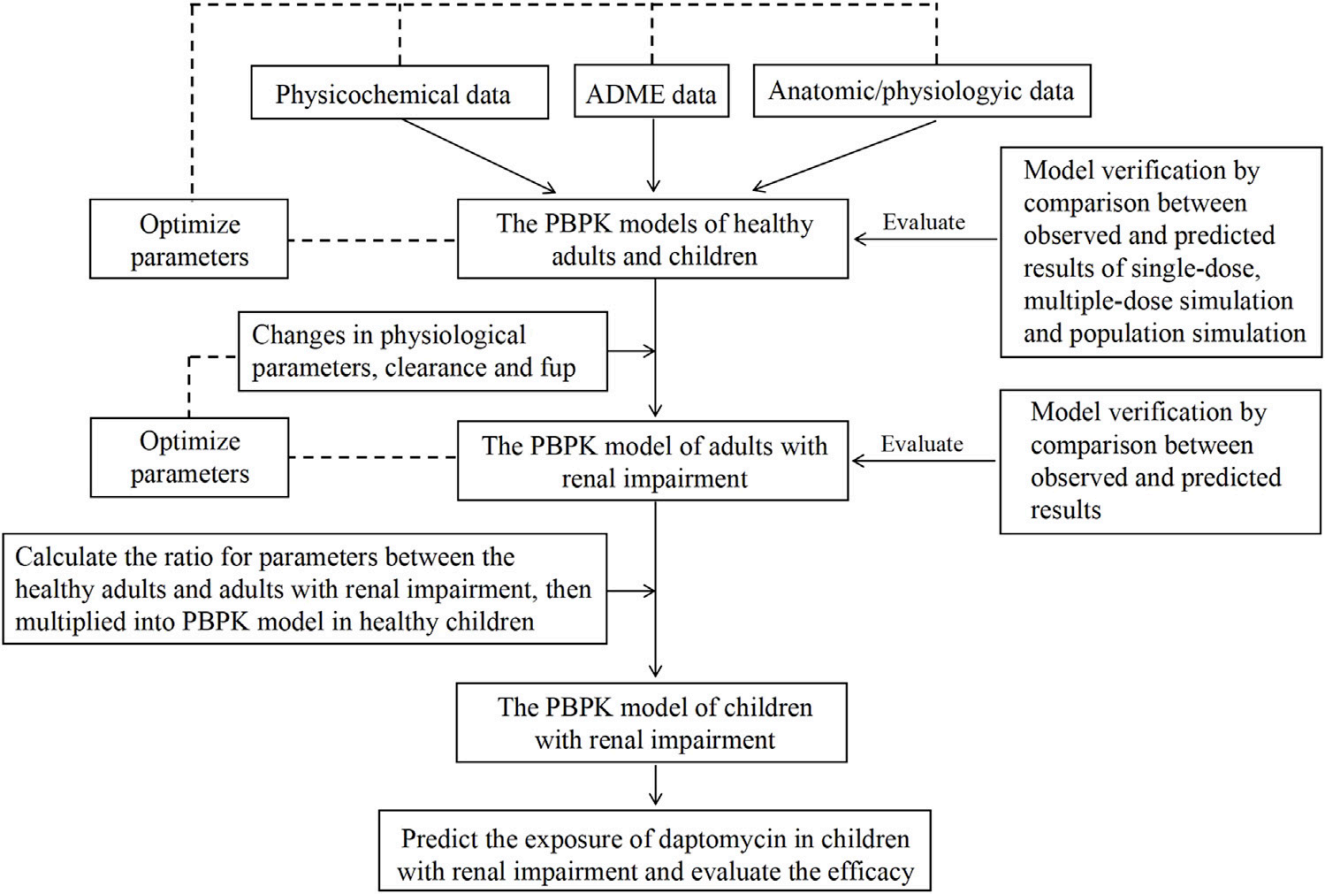
Workflow for PBPK modeling.

We followed the FDA guidance on PBPK model development and workflow in children to build our pediatric PBPK model (Figure 1) (Leong et al., 2012; Zhou et al., 2016; Dallmann et al., 2018). Initially, we combined physicochemical data of daptomycin with pharmacokinetic parameters, including absorption, distribution, metabolism, and elimination (ADME), along with anatomical and physiological data, to develop the PBPK models of healthy adults and healthy children, using Grastroplus^®^ (Version 9.7, Simulations Plus Inc., Lancaster, CA). In the PBPK models of the healthy population, we set the tissues type as permeability-limited because of the low volume of distribution of daptomycin. Then, we verified and optimized the models by comparing the predicted values with the observed values from the literature (Dvorchik et al., 2003; Benvenuto et al., 2006; Abdel-Rahman et al., 2008; Cohen-Wolkowiez et al., 2012; Bradley et al., 2014; Gregoire et al., 2019). After optimization, the finalized healthy adults PBPK model served as the basis for developing the PBPK model of adults with renal impairment. After verification and optimization using the same method described above, the ratio of each significant parameter between healthy adults and adults with renal impairment was calculated and used as a reference to develop a PBPK model for children with renal impairment based on the healthy children PBPK model. Lastly, we predicted the daptomycin exposure in children with renal impairment and evaluated the PD.

### PK data

Clinical PK data for intravenously administered daptomycin in a healthy population (adults and pediatric patients) and adults with renal impairment were derived from the literature (Dvorchik et al., 2003; Benvenuto et al., 2006; Abdel-Rahman et al., 2008; Cohen-Wolkowiez et al., 2012; Bradley et al., 2014; Gregoire et al., 2019). The observed concentration-time profiles were directly captured from figures by digitization (GetData Graph Digitizer 2.26).

### Healthy population PBPK model development

We initially developed a healthy-adult PBPK model by extracting concentration-vs-time data of intravenously administered daptomycin from PK studies of healthy adults (Dvorchik et al., 2003; Benvenuto et al., 2006). The PK of daptomycin was generally linear and its protein binding rate was concentration-independent. Therefore, the fraction of the unbound drug in plasma (fup) did not change in the model of the same health status, even if different dosages were administered. As daptomycin is primarily excreted as the unchanged drug in the urine (approximately 78%) and feces (approximately 6%), its clearance pathway was divided into renal and non-renal clearance. The non-renal clearance was estimated by subtracting the renal clearance from the total clearance. We assumed that the non-renal pathway was biliary excretion and classified it as liver clearance. Related parameters were optimized to ensure that the predicted and observed values were close or equal. Vital physicochemical parameters and critical *in vitro* data for daptomycin are presented in Table 1. Simulated population data are listed in Supplementary Tables S1, S2. The following simulated population characteristics were used: virtual ages of 18–65 years in adult subjects and 12–17 years, 7–11 years, 2–6 years, and 1 year in pediatric subjects; virtual weight based on age; 100 subjects, of which 50% were male.

**TABLE 1:** Physicochemical parameters, *In Vitro* and *In Vivo* data of daptomycin used in the simulations.

Parameter	Value	References
Molecular weight, g/mol	1,620.7	DrugBank^a^
logP^b^	−0.47	ALOGPS
	−9.4	ChemAxon
	1.2	Optimized
Solubility, mg/mL	17.89	Estimated by ADMET Predictor™
pH for solubbility	4.2	Estimated by ADMET Predictor™
pK_a_ ^c^	2.98 (Acid); 9.59 (Base)	ChemAxon
Fraction unbound in blood	0.08–0.1	Referance
Blood-to-plasma ratio	0.72	Estimated by ADMET Predictor™
	0.2	optimized
Plasma binding protein	Human serum albumin	Referance

a https://go.drugbank.com/drugs/DB0008

b log P: oil–water partition coefficients.

c pK_a_: acid dissociation constant.

### Physiological changes in populations with renal impairment

The kidney is one of the main organs responsible for drug elimination. Renal impairment decreases the renal clearance of drugs and their metabolites *via* changes in glomerular filtration, tubular secretion, reabsorption, or active transport (Zhang et al., 2009).

Typically, kidney clearance (CLr) is derived as follows:

$$CLr=CL_{filt}+{CL}_{\sec}-CL_{reabs}$$ (1)

where CL_filt_ is kidney filtration clearance, CL_sec_ is clearance by active and passive secretion, and CL_reabs_ is clearance by active and passive reabsorption.

When a drug is distributed to the kidneys, a fraction of the drug may be diverted to the kidney tubules by filtration. Based on PK studies, renal excretion is not the only elimination pathway of daptomycin, and its renal clearance is commonly described as a filtration process due to the absence of data showing a contribution of renal tubular secretion to the renal excretion of daptomycin. Therefore, we assumed that the non-renal pathway was biliary excretion and classified it as liver clearance:

$$CL=CL_{renal}+{CL}_{\mathrm{non-renal}}$$ (2)

$$CL=fup*GFR+CL_{H}$$ (3)

where CL_renal_ is renal clearance, CL_non-renal_ is non-renal clearance, and CL_H_ is liver clearance.

The renal function of the healthy population was defined as a glomerular filtration rate (GFR) > 90 ml/min per 1.73 m^2^. Renal impairment criteria included subjects with mild renal impairment (GFR 60 to<90 ml/min per 1.73 m^2^), moderate renal impairment (GFR 30 to<60 ml/min per 1.73 m^2^), severe renal impairment (GFR 15 to <30 ml/min per 1.73 m^2^), and end-stage renal disease (GFR <15 ml/min per 1.73 m^2^).

Although the most obvious effect of renal impairment is the change in renal excretion of drugs and their metabolites, it may also be associated with other changes, such as changes in absorption, plasma protein binding, and drug distribution (the USA, 2010).

### Scaling of the adult PBPK model to pediatric patients with renal impairment

The adult PBPK model was used to establish the daptomycin PBPK model in children. The algorithms implemented in GastroPlus were used to generate a virtual pediatric population of ages 1–17 years. The default physiological parameters implemented in GastroPlus were considered for the current model, and the children PBPK model was used to simulate the PK profiles in this special population.

All drug parameters (e.g., physicochemical properties) were fixed and only parameters related to physiology (e.g., body weight, blood flow, organ volumes, GFR, plasma protein binding levels, hematocrit, and cardiac output) were modified using the software in order to mimic the physiology of pediatrics.

Renal and non-renal clearance, as defined and quantified in the adult PBPK model, were scaled to the pediatric level. Briefly, renal clearance scaling was based on age-dependent GFR as the default setting. Non-renal clearance of daptomycin in the pediatric population was classified as liver clearance as well, and its scaling was based on the ratio of non-renal clearance to renal clearance in the adult PBPK model or children’s total clearance minusthe renal clearance. With the deterioration of renal function, the renal clearance changed based on the database in the GastroPlus software, while the non-renal clearance remained unchanged. Simulations with virtual populations of children from an age of one to adolescents aged 17 years were performed.

### Model validation

The PBPK model was assessed by calculating the fold error between the observed and predicted values. If the fold error was less than 2, the prediction was considered successful (Jones et al., 2006; Zhang et al., 2015).

Fold error = observed/predicted (observed value > predicted value) (Eq. 4)
Fold error = predicted/observed (observed value < predicted value) (Eq. 5)

### Dose optimization in children with renal impairment

We used the finalized pediatric PBPK model to predict the impact of renal impairment on the PK of daptomycin in pediatric patients and to evaluate optimal dosing for children using development-based age groups. Consistent with the concentration-dependent bactericidal activity of daptomycin, the ratio of the area under the plasma concentration-time curve to the minimum inhibitory concentration (AUC/MIC) represents a predictive parameter for the antibacterial efficacy of daptomycin. The antibiotic is highly protein-bound, and most researchers use AUC/MIC ≥666 as the PK-PD index of daptomycin (Falcone et al., 2013; Soraluce et al., 2018; Urakami et al., 2019; Wei et al., 2020; Yamada et al., 2020). Although some researchers consider the free, unbound portion of a drug as medicinally effective, and the *f*AUC/MIC is used as an evaluation criterion (Turnidge et al., 2020), we chose to examine the total daptomycin concentration.

### Monte-Carlo simulation

Daptomycin possesses substantial *in vitro* activity against aerobic Gram-positive cocci, including staphylococci, streptococci, and enterococci. Based on the EUCAST information (https://eucast.org/), the MIC values 0.125, 0.25, 0.5, 1, 2, 4, and 8 mg/L were used for Monte Carlo simulations, which were performed separately for each MIC. Since AUC/MIC ≥666 and ≥143 are commonly used as PK/PD indices of daptomycin for MRSA and *E. faecium*, respectively, we used them as a measure for treatment efficacy calculated *via* 10,000 randomly resampled subjects by using Monte-Carlo simulation (Crystal Ball software version 11.1.2.4.000).

## Results

### Development and evaluation of daptomycin PBPK models in healthy populations

The ADME properties of daptomycin drugs were determined using software simulations. Simulations of healthy adults and children were performed using the respective optimized PBPK models. PBPK models with substantial prediction ability were developed using vital physicochemical parameters and critical *in vitro* data of daptomycin, as presented in Table 1. The simulation results of single- and multiple-dose administration in healthy adults are shown in Supplementary Figure S1 and Supplementary Figure S5. The simulation results using 4 mg/kg in pediatric patients are shown in Supplementary Figure S2 and Figure 3. The healthy children population simulation results for a single dose based on the literature and for dosage regimens from the FDA-approved daptomycin label are shown in Supplementary Figure S3 and Supplementary Figure S4. The accuracy assessment of the predicted results is presented in Tables 2, 3. Remarkably, the predicted pharmacokinetic parameters were close to the observed values.

**TABLE 2:** Observed and simulated pharmacokinetic parameters of daptomycin after intravenous administration of different dosing regimens in adult.

Population	Physiological Status	Dose	Dosing interval	AUC_0-t_ (μg·h/mL)			C_max_ (μg/mL)		
				Observed	Predicted	Fold-error	Observed	Predicted	Fold-error
Healthy Adult									
(35 yr, 70 kg, BMI 25.71)	Healthy	6 mg/kg^c^	q24h	708.86	580	**1.22**	97.605	86.347	**1.13**
		8 mg/kg^c^		742.63	772.99	**1.04**	108.85	115.13	**1.06**
		10 mg/kg^c^		912.73	966.24	**1.06**	130.55	143.91	**1.10**
		12 mg/kg^c^		1180	1159.5	**1.02**	168.39	172.69	**1.03**
		4 mg/kg^d^		361.04	386.49	**1.07**	54.595	57.563	**1.05**
		6 mg/kg^d^		587.05	579.74	**1.01**	86.487	86.345	**1.00**
		8 mg/kg^d^		897.06	772.99	**1.16**	116.22	115.13	**1.01**
Adult with RI^a^									
(67 yr, 76 kg, BMI 25.27)	Healthy	10 mg/kg^e^	q24h	854.92	961.05	**1.12**	134.57	139.04	**1.03**
	Mild RI			940	1023.1	**1.09**	138.25	137.5	**1.01**
	Moderate RI			896.85	1087	**1.21**	105.55	122.54	**1.16**
	Severe RI		q48h	1403.9	1737.3	**1.24**	114.45	104.64	**1.09**
	ESRD^b^			-	1976.8	-	-	90.01	-

a RI: Renal Impairment.

b ESRD: End-Stage Renal Disease

c Benvenuto, M., Benziger, D.P., and Yankelev, S. (2006). Pharmacokinetics and tolerability of daptomycin at doses up to 12 milligrams per kilogram of body weight once daily in healthy volunteers. Antimicrob Agents Chemother. 50:3245-3249. doi: 10.1128/AAC.00247-06

d Dvorchik, B.H., Brazier, D., Debruin, M.F., and Arbeit, R.D. (2003). Daptomycin pharmacokinetics and safety following administration of escalating doses once daily to healthy subjects. Antimicrob Agents Chemother. 47:1318-1323. doi: 10.1128/AAC.47.4.1318-1323.2003

e Gregoire, N., Marchand, S., Ferrandiere, M., Lasocki, S., Seguin, P., Vourc'h, M., et al. (2019). Population pharmacokinetics of daptomycin in critically ill patients with various degrees of renal impairment. Journal of Antimicrobial Chemotherapy. 74:117-125. doi: 10.1093/jac/dky374

q24h: once every 24 h, q48h: once every 48 h.

The bold values indicated as emphasis and highlight.

**TABLE 3:** Observed and simulated pharmacokinetic parameters of daptomycin after intravenous administration of different dosing regimens in healthy child.

Population	Physiological Status		Age (yr)	Dose	Dosing interval	AUC_0-t_ (μg·h/mL)			C_max_ (μg/mL)		
	Age (yr)/Weight (kg)/BMI					Observed	Predicted	Fold-error	Observed	Predicted	Fold-error
Child	Healthy	15/70.6/22.8	12–17	4 mg/kg^a^	q24h	374.4	386.42	**1.03**	50	54.24	**1.08**
		10/39.7/19.1	7–11			271	282.25	**1.04**	48	52.08	**1.08**
		5/18/15.68	2–6			215.3	194.92	**1.10**	43.8	47.15	**1.08**
		15/70.6/22.8	12–17	5 mg/kg^b^		434	487.16	**1.12**	76.4	68.38	**1.12**
		10/39.7/19.1	7–11	7 mg/kg^b^		543	490.41	**1.11**	92.4	90.49	**1.02**
		5/18/15.68	2–6	9 mg/kg^b^		452	438.36	**1.03**	90.3	90.01	**1.00**
		1/10.23/17.1	1–2	10 mg/kg^b^		462	528.11	**1.14**	81.6	97.47	**1.19**
		15/70.6/22.8	12–17	7 mg/kg^b^		656	764.9	**1.17**	104	104.07	**1.00**
		10/39.7/19.1	7–11	9 mg/kg^b^		579	657.3	**1.14**	104	119.37	**1.15**
		5/18/15.68	1–6	12 mg/kg^b^		620	598.1	**1.04**	106	120.91	**1.14**

a Abdel-Rahman, S.M., benziger; D.P., jacobs; R.F., jafri; H.S., hong; E.F., and Kearns, G.L. (2008). Single-dose pharmacokinetics of daptomycin in children with suspected or proved Gram-positive infections. Pediatr Infect Dis J. 27:330–334. Doi: 10.1097/INF.0b013e318160edfc.

b FDA-Label-daptomycin. Available at: https://www.accessdata.fda.gov/drugsatfda_docs/label/2021/021572s065s066lbl.pdf

The bold values indicated as emphasis and highlight.

**FIGURE 2:**
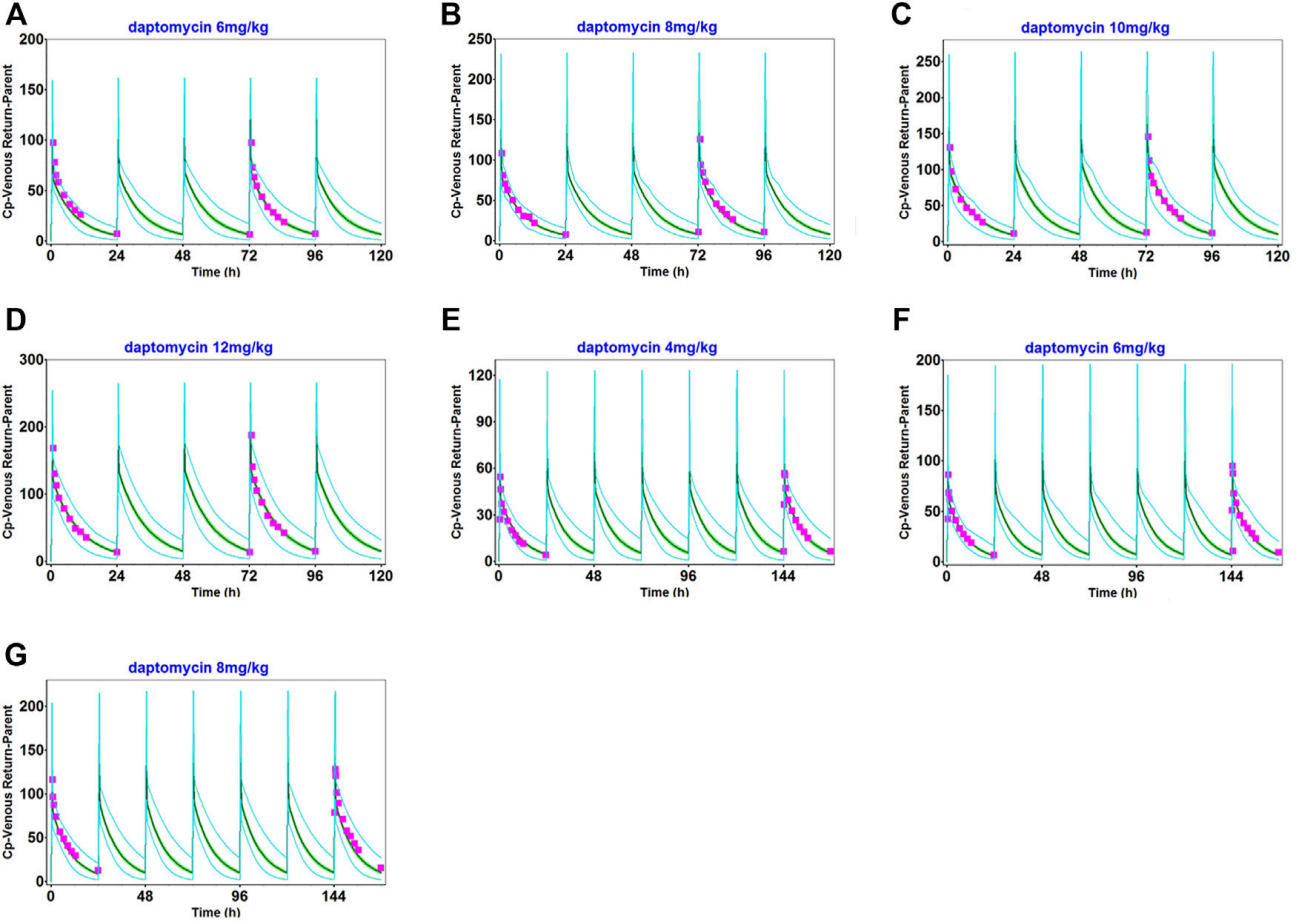
Population simulation of daptomycin after administering 6 mg/kg **(A)**, 8 mg/kg **(B)**, 10 mg/kg **(C)**, and 12 mg/kg **(D)** as multiple intravenous doses for 5 days in healthy adults. 4 mg/kg **(E)**, 6 mg/kg **(F)**, and 8 mg/kg **(G)**, as multiple intravenous doses for 7 days in healthy adults. The shaded area represents the 90% confidence interval for the simulated data, the blue lines indicate the corresponding drug concentration-time curves with a 95% probability, and the red squares represent the daptomycin concentrations derived from the literature.

**FIGURE 3:**
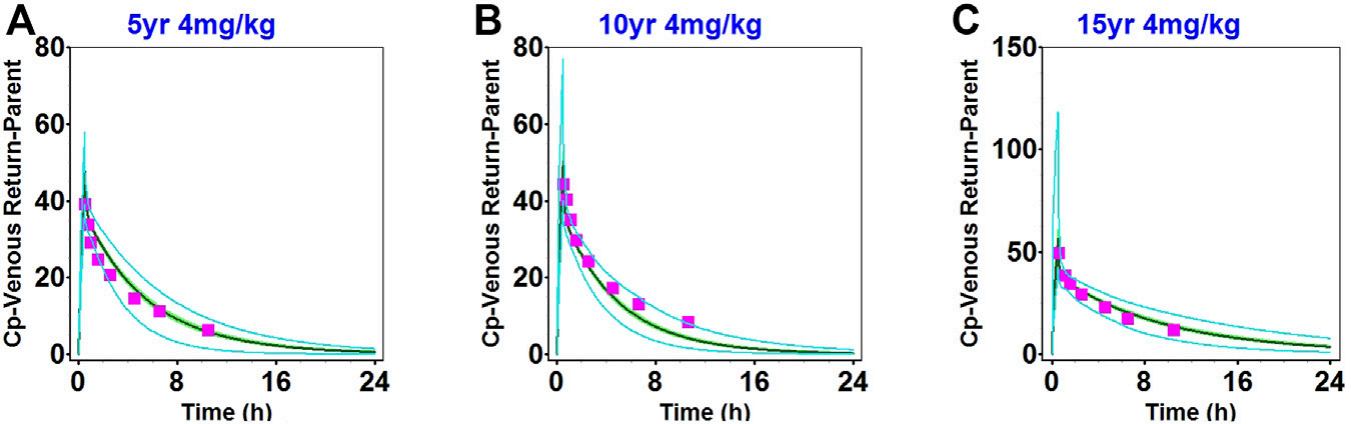
Population simulation of daptomycin after administering 4 mg/kg as a single intravenous dose in 2–6-year-old healthy children **(A)**, 7–11-year-old healthy children **(B)**, and 12–17-year-old healthy children **(C)**. The shaded area represents the 90% confidence interval for the simulated data, the blue lines indicate the corresponding drug concentration-time curves with a 95% probability, and the red squares represent the daptomycin concentrations derived from the literature.

The observed clinical values are shown along the simulated mean plasma concentration-time profiles derived from population simulations presented in Figures 2, 3, and Supplementary Figure S6. The strong agreement between predicted and observed drug concentration-time profiles indicated that this model accurately captured the ADME properties of daptomycin.

### Establishment and validation of the daptomycin PBPK model in adults with renal impairment

**FIGURE 4:**
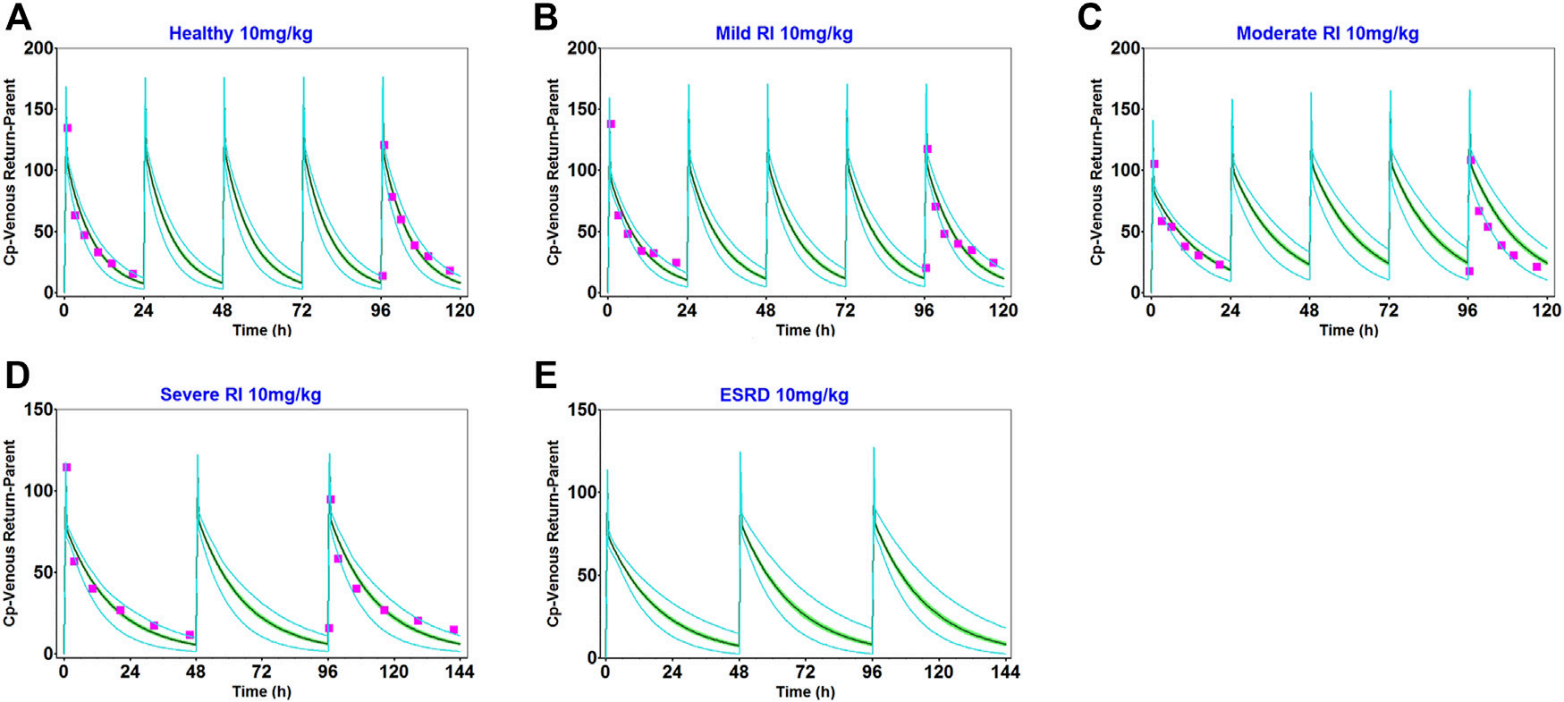
Population simulation of daptomycin after administering 10 mg/kg as multiple intravenous doses in healthy adults **(A)**, adults with mild renal impairment **(B)**, and adults with moderate renal impairment **(C)** for 5 days. In adults with severe renal impairment **(D)** and adults with end-stage renal disease **(E)** for 6 days. The shaded area represents the 90% confidence interval for the simulated data, the blue lines indicate the corresponding drug concentration-time curves with a 95% probability, and the red squares represent the daptomycin concentrations derived from the literature.

The main physiological changes associated with renal impairment were incorporated into a disease-modified model to derive predictions of daptomycin after single- and multiple-dose administrations of 10 mg/kg, which were compared with clinical pharmacokinetic data obtained from patients with different degrees of renal impairment (Supplementary Figures S7, S8). The population simulation results are shown in Figure 4. The visual predictive checks indicated that the established model performed well in predicting daptomycin concentrations in adult patients with renal impairment.

We obtained fold error values of approximately 1.24 times or less by comparing the simulated AUC and C_max_ results with the observed values (Table 2). Based on the 2-fold error threshold, the fold error values of AUC and C_max_ indicated that the simulation was consistent with the observed values. Thus, these results demonstrated that the disease-modified PBPK model correctly simulated daptomycin exposure in adult patients with renal impairment, which provided a strong platform for performing simulations of daptomycin treatment in pediatric patients with renal impairment.

### Prediction of the daptomycin concentration in pediatric patients with renal impairment

We simulated the daptomycin concentrations in pediatric patients with different degrees of renal impairment after intravenous administration of 4–12 mg/kg and predicted the patients’ plasma concentration-time profiles of daptomycin. Similar to the results for adults with different degrees of renal function, the C_max_ and AUC of daptomycin in children with mild-to-moderate renal impairment did not significantly differ from those in healthy children. The AUC increased by an average of 1.55-fold and 1.85-fold in severe renal impairment and end-stage renal disease, respectively (Table 4), and changes were predicted to be more pronounced in younger children. Based on the changes in AUC, no dosage adjustment was needed in pediatric patients with mild-to-moderate renal impairment. According to the type of infection, the dosage adjustment recommendations for each age group are detailed in Table 6. These results will have significant implications for predicting drug efficacy and adverse drug reactions of clinical-dosage regimens.

**TABLE 4:** Simulated pharmacokinetic parameters of daptomycin after intravenous administration of different dosing regimens in pediatric patient with renal impairment.

Population	Physiological Status		Age (yr)	Dose	Predicted AUC_0-24h_ (μg·h/mL)	Ratio (RI/Healthy)	Predicted C_max_ (μg/mL)	Ratio (RI/Healthy)
	Age (yr)/Weight (kg)/BMI							
Child	Healthy	15/70.6/22.8	12–17	5 mg/kg q24h	487.16		68.38	
	Mild RI^a^				478.45	**0.98**	63.68	**0.93**
	Moderate RI			5 mg/kg q48h	526.88	**1.08**	56.81	**0.83**
	Severe RI				603.72	**1.24**	48.83	**0.71**
	ESRD^b^				650.29	**1.33**	42.24	**0.62**
	Healthy	10/39.7/19.1	7–11	7 mg/kg q24h	490.41		90.49	
	Mild RI				492.74	**1.00**	84.07	**0.93**
	Moderate RI				583.18	**1.19**	75.63	**0.84**
	Severe RI			7 mg/kg q48h	729.77	**1.49**	66.76	**0.74**
	ESRD				848.82	**1.73**	58.99	**0.65**
	Healthy	5/18/15.68	2–6	9 mg/kg q24h	438.36		90.01	
	Mild RI				446.94	**1.02**	82.81	**0.92**
	Moderate RI				560.18	**1.28**	75.36	**0.84**
	Severe RI			9 mg/kg q48h	762.94	**1.74**	70.08	**0.78**
	ESRD				963.57	**2.20**	64.72	**0.72**
	Healthy	1/10.23/17.1	1–2	10 mg/kg q24h	528.11		97.47	
	Mild RI				550.29	**1.04**	89.21	**0.92**
	Moderate RI				679.98	**1.29**	80.6	**0.83**
	Severe RI			10 mg/kg q48h	906.76	**1.72**	75.99	**0.78**
	ESRD				1125.8	**2.13**	71.36	**0.73**
	Healthy	15/70.6/22.8	12–17	7 mg/kg q24h	683.13		95.89	
	Mild RI				670.91	**0.98**	89.3	**0.93**
	Moderate RI				738.83	**1.08**	79.67	**0.83**
	Severe RI			7 mg/kg q48h	846.58	**1.24**	68.47	**0.71**
	ESRD				911.89	**1.33**	59.22	**0.62**
	Healthy	10/39.7/19.1	7–11	9 mg/kg q24h	629.77		116.2	
	Mild RI				632.77	**1.00**	107.95	**0.93**
	Moderate RI				748.9	**1.19**	97.12	**0.84**
	Severe RI			9 mg/kg q48h	937.15	**1.49**	85.73	**0.74**
	ESRD				1090	**1.73**	75.75	**0.65**
	Healthy	5/18/15.68	1–6	12 mg/kg q24h	584.48		120.01	
	Mild RI				595.92	**1.02**	110.41	**0.92**
	Moderate RI				746.9	**1.28**	100.48	**0.84**
	Severe RI			12 mg/kg q48h	1017.3	**1.74**	93.44	**0.78**
	ESRD				1284.8	**2.20**	86.29	**0.72**

a RI, renal impairment.

b ESRD, End-Stage Renal Disease.

The bold values indicated as emphasis and highlight.

### Pharmacodynamic evaluation of daptomycin in pediatric patients with renal impairment

**FIGURE 5:**
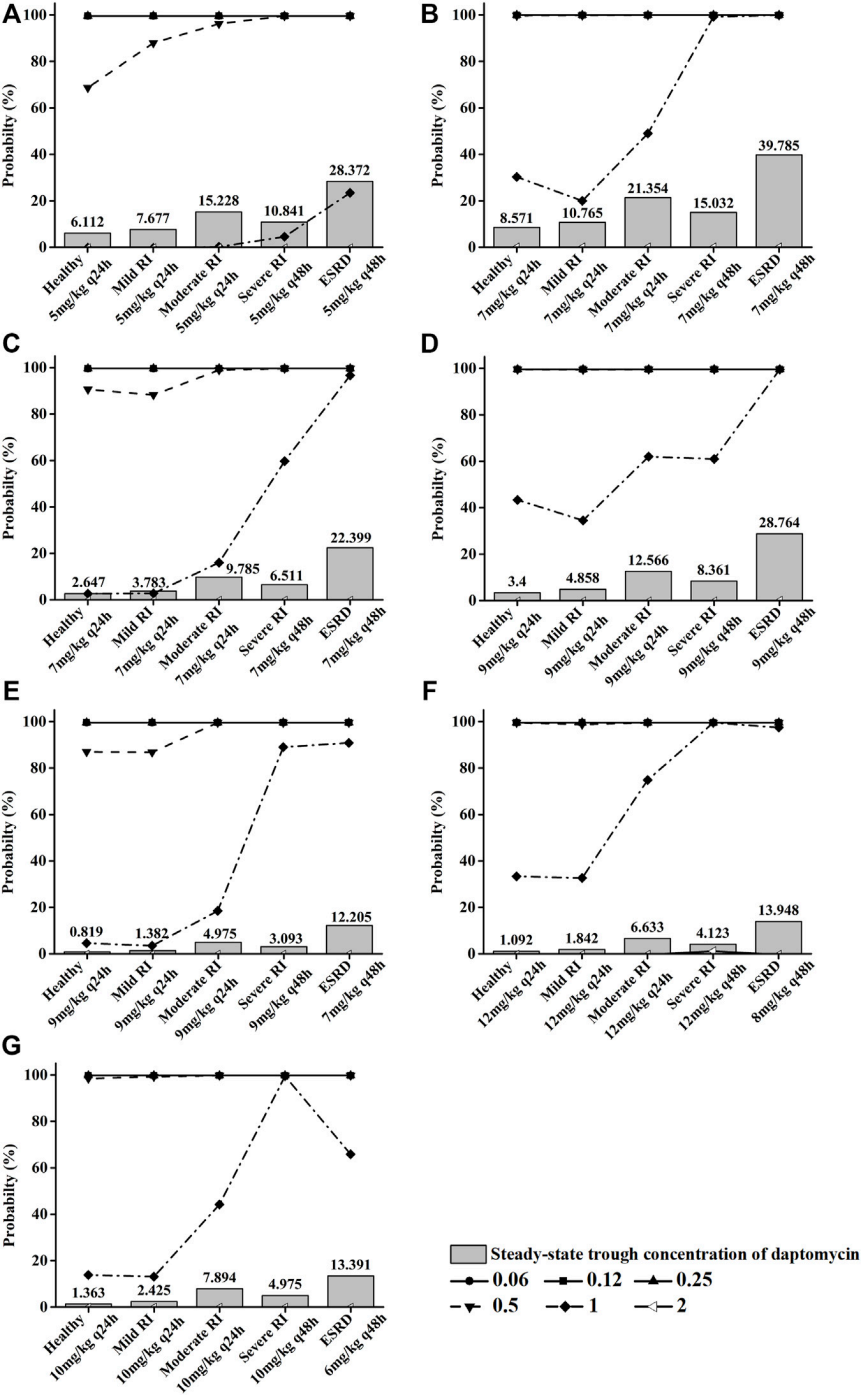
Pictures **(A) (B)**, **(C) (D)**, **(E) (F)**, and **(G)** show the PTA for different MIC values and steady-state trough concentrations of daptomycin, respectively, when children aged 12–17 years, 7–11 yeaears, 2–6 years, and 1 year were given different recommended doses at different renal function states. Each symbol indicates MIC (mg/L).

In this study, we performed Monte Carlo simulations using the combination of the Crystal Ball software (version 11.1.2.4.000) and population simulation from GastroPlus^®^. Based on the daptomycin MIC_90_ values from EUCAST (Supplementary Tables S1, S2) and AUC/MIC ≥666 or AUC/MIC ≥143 as the commonly used PK/PD index of daptomycin for MRSA or *E. faecium* (Wei et al., 2020), the antibiotic exhibited an excellent antibacterial activity at MIC ≤0.5 mg/L. We assessed the probability of target attainment (PTA) and steady-state trough concentrations of daptomycin at different renal function doses (Figure 5) combined with antimicrobial efficacy and the risk of rhabdomyolysis occurrence to comprehensively evaluate pharmacodynamics. When MIC ≤0.5 mg/L, the PTA of the recommended dose can almost reach higher than 90% in different renal function states; at an MIC of 1 mg/L, PTA increased with the deterioration of renal function; at an MIC of 2 mg/L, there was almost no antibacterial effect. The steady-state trough concentration of daptomycin also generally increased with worsening renal function, indicating an increased risk of rhabdomyolysis. Using the CFR results for MRSA or *E. faecium*, the recommended dose for *S. aureus* bacteremia on the FDA label and the adjusted dose derived from our PBPK model could reach more than 90% in healthy children as well as in pediatric patients with different degrees of renal impairment. Furthermore, the pharmacodynamic evaluation for dose adjustment based on the AUC in pediatric patients with renal impairment also generated satisfactory results (Table 5).

**TABLE 5:** Cumulative fraction of response expectation values (%) against methicillin-resistant *S. aureus* (MRSA) and *Enterococcus faecium (E. faecium)* for each daptomycin dosing regimen in pediatric patients.

Patient population	Dose	Cumulative fraction of response (%)									
		Dosage regimens against MRSA					Dosage regimens against E.faecium				
		Healthy	Mild RI^a^	Moderate RI	Severe RI^*c*^	ESRD^b^ ^.^ ^c^	Healthy	Mild RI	Moderate RI	Severe RI^c^	ESRD^*c*^
12–17 years	**5 mg/kg q24 h**	70.49	80.99	85.32	87.93	90.42	71.98	77.49	79	86.19	96.94
7–11 years	**7 mg/kg q24 h**	82.72	81.19	88.92	94.86	99.69	78.73	78.61	86.2	97.03	99.12
2–6 years	**9 mg/kg q24 h**	81.05	80.78	89.7	98.71	98.84 (7 mg/kg)	78.07	77.38	89.63	98.37	98.36 (7 mg/kg)
1–2 years	**10 mg/kg q24 h**	88.05	89.36	93.23	99.95	95.29 (6 mg/kg)	84.8	86.07	94.15	98.41	98.31 (6 mg/kg)
12–17 years	**7 mg/kg q24 h**	91.38	89.87	93.45	99.87	100	90.39	88.55	96.54	98.54	98.35
7–11 years	**9 mg/kg q24 h**	92.95	83.7	95.39	95.03	100	93.12	78.73	97.26	97	98.87
1–6 years	**12 mg/kg q24 h**	91.4	90.73	96.94	100	99.74 (8 mg/kg)	91.41	89.44	97.63	98.86	98.33 (8 mg/kg)

a RI, renal impairment.

b ESRD, End-Stage Renal Disease.

c The interval of administration was every 48 h.

The bold values indicates emphasis and highlight.

## Discussion

The primary objective of this study was to predict the PK of daptomycin in pediatric patients with renal impairment by developing PBPK models. The resulting PK simulations address a lack of reports on the use of daptomycin in those populations owing to the difficulties in conducting clinical daptomycin PK studies in children with renal impairment for various reasons, including ethical restrictions.

We established and optimized the basic PBPK model of daptomycin in healthy populations. Our modeling approach generated excellent simulation results for both healthy adults and healthy children based on the fold error values of less than 1.24. During the modeling process, renal and non-renal elimination pathways were incorporated into the daptomycin model. Because the non-renal elimination pathway has not been clearly established, we assumed that it occurred *via* biliary excretion, which we considered as liver clearance. Renal daptomycin clearance is commonly described as a filtration process owing to a lack of data showing the contribution of transporters to renal excretion in humans, and the non-renal clearance was estimated by subtracting the renal clearance from the total clearance. This model optimization method is similar to the published vancomycin PBPK modeling approach (Emoto et al., 2018). Studies also report that daptomycin is subjected to bile excretion, which shows that our hypothesis is reasonable (Tascini et al., 2011).

Owing to the limited pharmacokinetic studies of daptomycin in children, we only obtained specific data points measured at a dose of 4 mg/kg (Abdel-Rahman et al., 2008). To circumvent this lack of published data, we incorporated various recommended dosing regimens for different age groups from the FDA label into the model and compared the predicted values with the FDA data to further verify the reliability of the model (FDA, 2020). Remarkably, our prediction results met the 2-fold error threshold criterion with a mean error fold of 1.09. The daptomycin clearance, V_ss,_ and half-life (T_half_) in our model are similar to those reported in the literature (Supplementary Table S3). In the group comprising the youngest children, the clearance rate was higher and the T_half_ was shorter than in the groups with older children. The trend for these two parameters was consistent with the results reported by other research groups (FDA, 2020; Abdel-Rahman et al., 2008; Durand et al., 2014). In summary, our model was validated by comparing various pharmacokinetic parameters.

Remarkably, although the GFR decreased in our model with the decline of renal function, the biliary excretion did not change, which is also consistent with the reported assessment by Nolin et al. (2008). In contrast, some reports indicatethat bile excretion changes when renal function declines (Nolin et al., 2008; Schijvens et al., 2020). The controversy on this aspect may require further research. We hypothesize that this may be related to the specificity of the drug and the expression of the transporter involved in the excretion pathway. Therefore, the biliary excretion in our model remained unchanged to obtain predicted values that were closer to the measured data. The fup value changed only slightly, with 8% in healthy condition, 9% in mild renal impairment, and 10% in moderate renal impairment to end-stage renal disease, which is the same as previously reported (Gregoire et al., 2019). This change made not only the simulated total plasma concentration closer to the measured value, but also made the predicted free concentration, which is related to fup, closer to the measured value. However, in the PBPK model of adults with renal impairment, we corrected the fup to 9% in healthy status to make the prediction result closer to the measured value because the included population had an average median age of approximately 67 years, and the serum albumin is generally low in the older population (Gom et al., 2007). We made assumptions on the Blood-to-plasma ratio (RBP) of daptomycin based on the following information: Daptomycin is a highly protein-bound antibacterial drug, mainly bound to serum albumin, so its drug concentration in plasma is much higher than that in whole blood. Furthermore, because of its low volume of distribution, of only 0.1 L/kg and about 7 L in adults, it is mainly distributed in the plasma, and to a lower extent in the extracellular water space fluid. Given its extremely hydrophilic nature, it is very unlikely that daptomycin has the ability to penetrate human cells in significant amounts (Thallinger et al., 2008). Therefore it has a low potential to penetrate blood cells. In contrast to daptomycin, several hundred liters are the distribution volumes of the macrolides class of antibiotics such as azithromycinfor which the RBP was estimated to be 1 (Johnson et al., 2016; Guimarães et al., 2021). On the other hand, it is possible that we used 1.2 as optimized logP value, which might be quite a bit higher than experimental, resulting in the need to use a lower RBP to satisfy the daptomycin distribution. Further study is required on this front.

In this study, we performed Monte Carlo simulations using the combination of Crystal Ball software and population simulation from GastroPlus^®^. This method combines human parameters in a physiological-based population simulation with mathematical-statistical methods to yield results compatible with the real world. The optimized dosing recommendations (Table 6) for daptomycin in pediatric patients with renal impairment could potentially achieve the same AUC values as those in healthy children. For each dosage regimen, 10,000 random simulations were performed to obtain the PTA and CFR predictions (Figure 5 and Table 5). Based on the CFR results, the recommended dose for *S. aureus* bacteremia on the FDA label and the adjusted dosage derived from our PBPK model can reach a CFR of more than 90% for MRSA or *E. faecium*. A comparison with the published Monte Carlo simulation data of different daptomycin doses in children indicated that our PTA and CFR data for healthy children were similar (Wei et al., 2020). The similarity of these data also increases the credibility of our PBPK model.

**TABLE 6:** Recommended dose adjustments for daptomycin in children with renal impairment.

	Infection	Age (yr)	Healthy children	Mild RI^a^	Moderate RI^a^	Severe RI^a^	ESRD^b^
Proposed recommended dose	**cSSSI** ^c^	12–17	5 mg/kg q24 h	no dosage adjustment need		5 mg/kg q48 h	5 mg/kg q48 h
		7–11	7 mg/kg q24 h			7 mg/kg q48 h	7 mg/kg q48 h
		2–6	9 mg/kg q24 h			9 mg/kg q48 h	7 mg/kg q48 h
		1–2	10 mg/kg q24 h			10 mg/kg q48 h	6 mg/kg q48 h
	**S. aureus Bacteremia** ^d^	12–17	7 mg/kg q24 h	no dosage adjustment need		7 mg/kg q48 h	7 mg/kg q48 h
		7–11	9 mg/kg q24 h			9 mg/kg q48 h	9 mg/kg q48 h
		1–6	12 mg/kg q24 h			12 mg/kg q48 h	8 mg/kg q48 h

a RI, renal impairment.

b ESRD, End-Stage Renal Disease.

c cSSSI, complicated skin and skin structure infections.

d *S. aureus* Bacteremia: *Staphylococcus aureus* Bloodstream Infections.

Elevation of creatinine phosphokinase (CPK) levels is an adverse reaction of daptomycin, and the severe form can develop into rhabdomyolysis (FDA, 2020). The increase in CPK is associated with high-dose daptomycin treatment and a declining renal function (Tai et al., 2018). Additionally, the plasma trough concentration (C_min_) of daptomycin ≥24.3 mg/L is reportedly associated with elevated CPK levels (Bhavnani et al., 2010). This adverse reaction should be especially considered for children with renal impairment. Therefore, we simulated the C_min_ at steady state in children with different renal functions under the adjusted daptomycin dose and evaluated the safety of the adjustment to a certain extent (Figure 5). The administration instructions indicate that a healthy population can reach a steady state after the third dose, but a decline in renal function prolongs the time for the drug concentration to reach a steady state correspondingly. In children aged 7–17 years with an onset of severe renal impairment, the steady state was attained after the fourth dose (q48 h), and in end-stage renal disease, the fifth dose (q48 h) was administered before reaching a steady state. Four daptomycin doses (q48 h) were needed to reach the steady state in children aged 1–6 years with severe renal impairment or end-stage renal disease. Clearance was higher in younger children than in older children, decreasing from around 20 ml/h/kg to around 11 ml/h/kg as age increased (Supplementary Table S3). Even if a higher dose was used in the younger age group, the steady-state trough concentration was far less than 24.3 mg/L. Furthermore, in children aged 7–11 and 12–17 years with end-stage renal disease, the daptomycin steady-state trough concentration was slightly higher than 24.3 mg/L, indicating that when renal function deteriorated, even if the dosage is adjusted, the CPK level should be closely monitored more than once a weekto prevent the occurrence of rhabdomyolysis (Kullar et al., 2014).

This study had several limitations. Firstly, there were no data sets available for daptomycin in pediatric patients with renal impairment to validate the model. Thus, for model improvement, it will be necessary to accumulate new clinical data. Secondly, daptomycin is not recommended in pediatric patients younger than 1 year of age because of the risk of potential adverse effects on the muscular, neuromuscular, and nervous systems observed in neonatal dogs (FDA, 2020). Although some scholars have conducted related studies on this population, we currently have no plans to establish a PBPK model for children less than 1 year old. In the future, we will use new research results as they become available for supplementing our model. Lastly, the MIC distributions of the pathogen isolates were obtained from EUCAST, which represents Europe but no other areas. However, compared with related PD studies (Falcone et al., 2013; Soraluce et al., 2018; Urakami et al., 2019; Wei et al., 2020; Yamada et al., 2020), the data did not differ substantially, and an impact on the results is not expected. Thus, our study can fill the gap in clinical data to a certain extent.

## Conclusion

In summary, we established, validated, and optimized PBPK models of daptomycin for healthy adults, healthy children, and adults with impaired renal function, which were used to derive and construct PBPK models for children with renal impairment. We used PBPK modelingto evaluate the pharmacokinetic changes of daptomycin in this special population and create adjusted dosing regimens. After evaluating the PD and adverse reactions, we obtained the recommended daptomycin doses for children with renal impairment. In pediatric patients with mild-to-moderate renal impairment, no dosage adjustments were needed. In pediatric patients with severe renal impairment, the dosing interval should be extended (from q24 h to q48 h). In patients with end-stage renal disease combined with cSSSI, the daptomycin dose of 9 mg/kg q24 h should be adjusted to 7 mg/kg q48 h in children aged 2–6 years, and 10 mg/kg q24 h should be adjusted to 6 mg/kg q48 h in children aged 1–2 years. For *S. aureus* bacteremia, 12 mg/kg q24 h should be adjusted to 8 mg/kg q48 h in children aged 1–6 years. Importantly, CPK should be closely monitored more than once a weekin pediatric patients with renal impairment. Our model may serve as a useful tool for predicting the PK of daptomycin and support dose adjustments or other relevant decisions in clinical settings.

## Data Availability

The original contributions presented in the study are included in the article/Supplementary Material, further inquiries can be directed to the corresponding author.
