# Supplementary material for: Physiologically based pharmacokinetic modeling of daptomycin dose optimization in pediatric patients with renal impairment
Source: Front Pharmacol. 2022 Aug 16;13:838599. doi: 10.3389/fphar.2022.838599 (PMC9424659; doi:10.3389/fphar.2022.838599)
Supplement: Supplementary file 1 [file DataSheet1.docx]

**Supplementary figures and tables**

**Article title:** Physiologically Based Pharmacokinetic Modelling of Daptomycin Dose Optimization in Pediatric Patients with Renal Impairment

**Journal name:**

**Author names:** Lingling Ye, Xiang You, Jie Zhou, Chaohui Wu, Meng Ke, Wanhong Wu, Pinfang Huang, Cuihong Lin*

**Corresponding author:** Cuihong Lin, Department of Pharmacy, the First Affiliated Hospital of Fujian Medical University, 20 Cha Zhong M. Rd, Fuzhou 350005, People’s Republic of China. E-mail: [lincuihong1974@sina.com](mailto:lincuihong1974@sina.com)

**
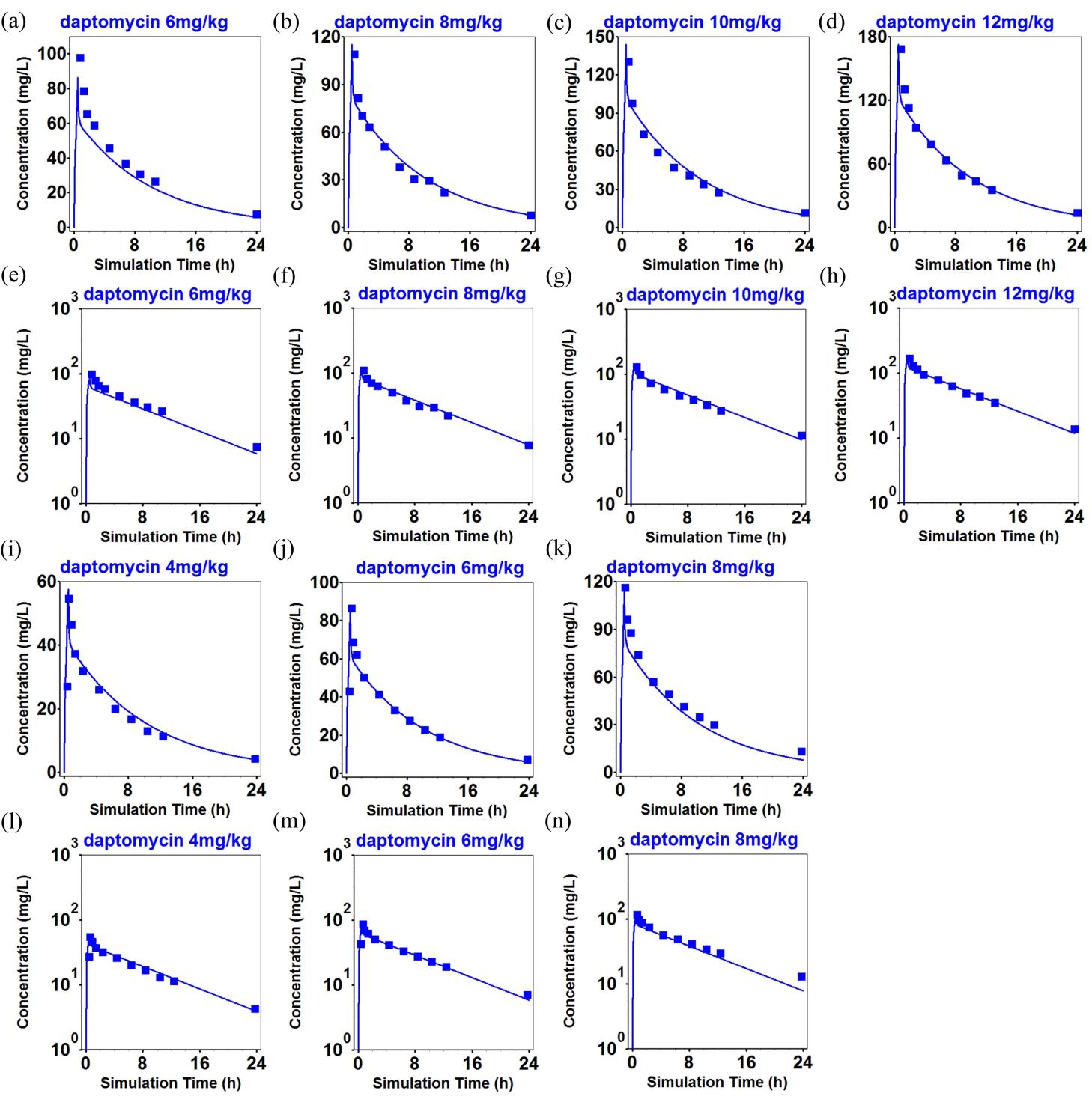
**

**Figure S1.** Mean drug concentration-time profiles of daptomycin predicted after administering 6 mg/kg (**a, e**), 8 mg/kg (**b, f**), 10 mg/kg (**c, g**), and 12 mg/kg (**d, h**), and observed after administering 4 mg/kg (**i, l**), 6 mg/kg (**j, m**), and 8 mg/kg (**k, n**) based on the literature,^27,28^ as a single intravenous dose in healthy adults. The solid squares represent the observed values. The solid thick lines represent the predicted value.

**
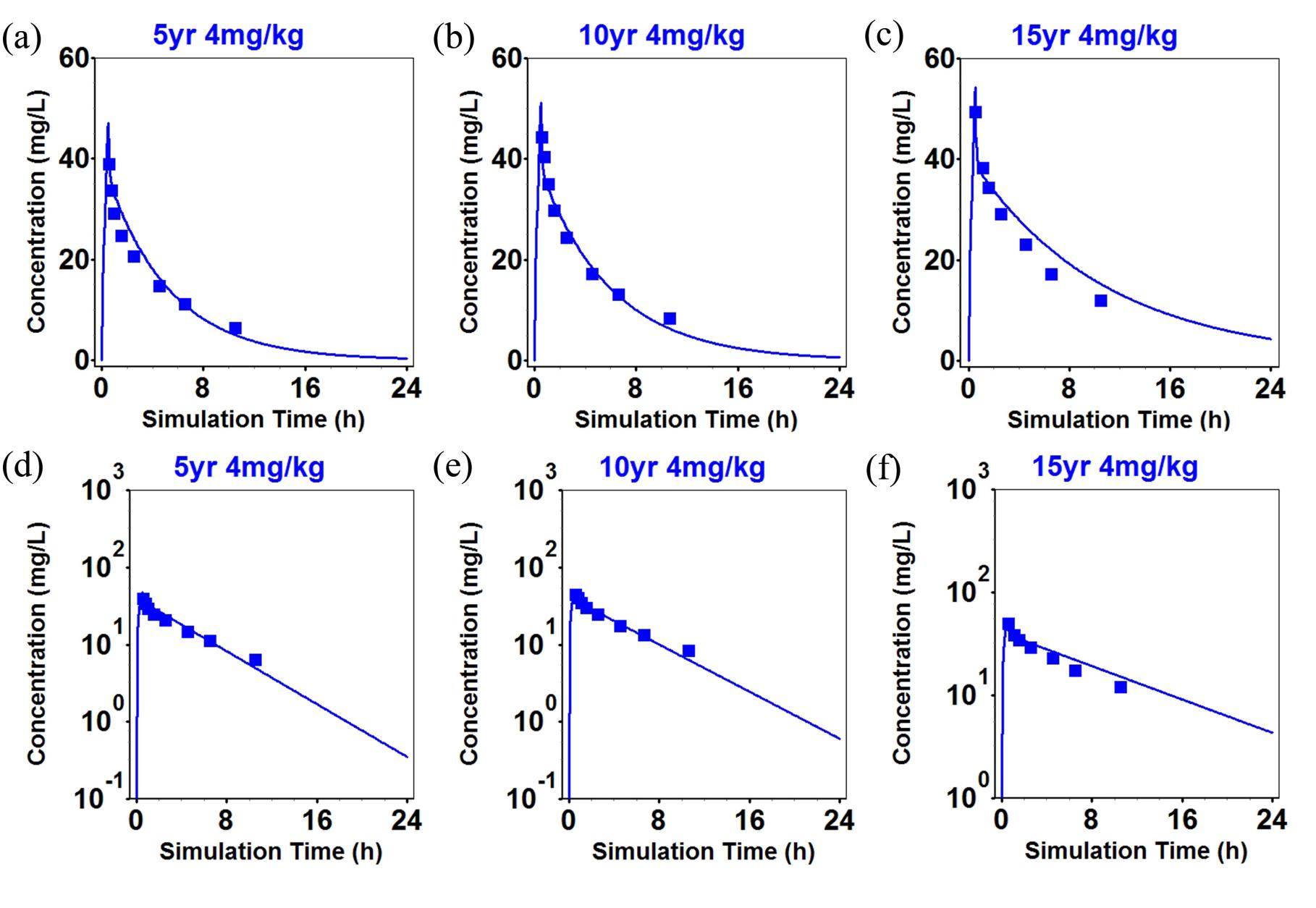
**

**Figure S2.** Mean drug concentration-time profiles of daptomycin after administering 4 mg/kg as a single intravenous dose in healthy children aged 12–17 years (**a, d**), healthy children aged 7–11 years (**b, e**), and healthy children aged 2–6 years (**c, f**). The solid squares represent the observed values.^32^The solid, thick lines represent the predicted values.

**
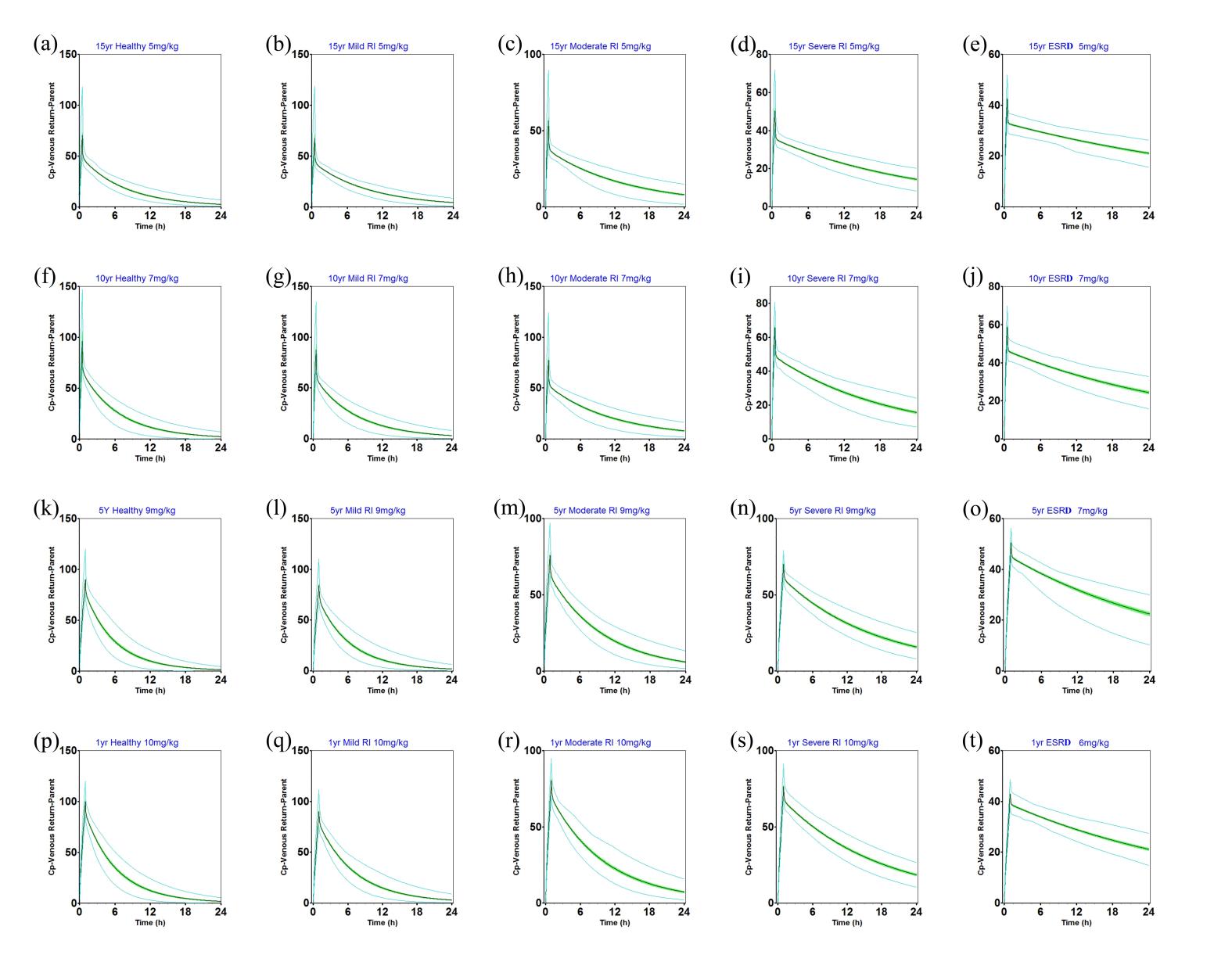
**

**Figure S3.** Population simulation of daptomycinafter administering 5 mg/kg as a single intravenous dose in healthy children aged 12–17 years (**a**) and pediatric patients with various degrees of renal impairment (**b, c, d, e**). 7 mg/kg as a single intravenous dose in healthy children aged 7–11 years (**f**) and pediatric patients with various degrees of renal impairment (**g, h, i, j**). 9 mg/kg as a single intravenous dose in healthy children aged 2–6 years (**k**) and pediatric patients with various degrees of renal impairment (**l, m, n, o**). 10 mg/kg as a single intravenous dose in healthy children aged 1–2 years (**p**) and pediatric patients with various degrees of renal impairment (**q, r, s, t**). The shaded area represents a 90% confidence interval for the simulated data, and the blue lines indicate the corresponding drug concentration-time curves with a 95% probability. The dosage is from the FDA drug insert for children with complicated skin and skin structure infections.

**
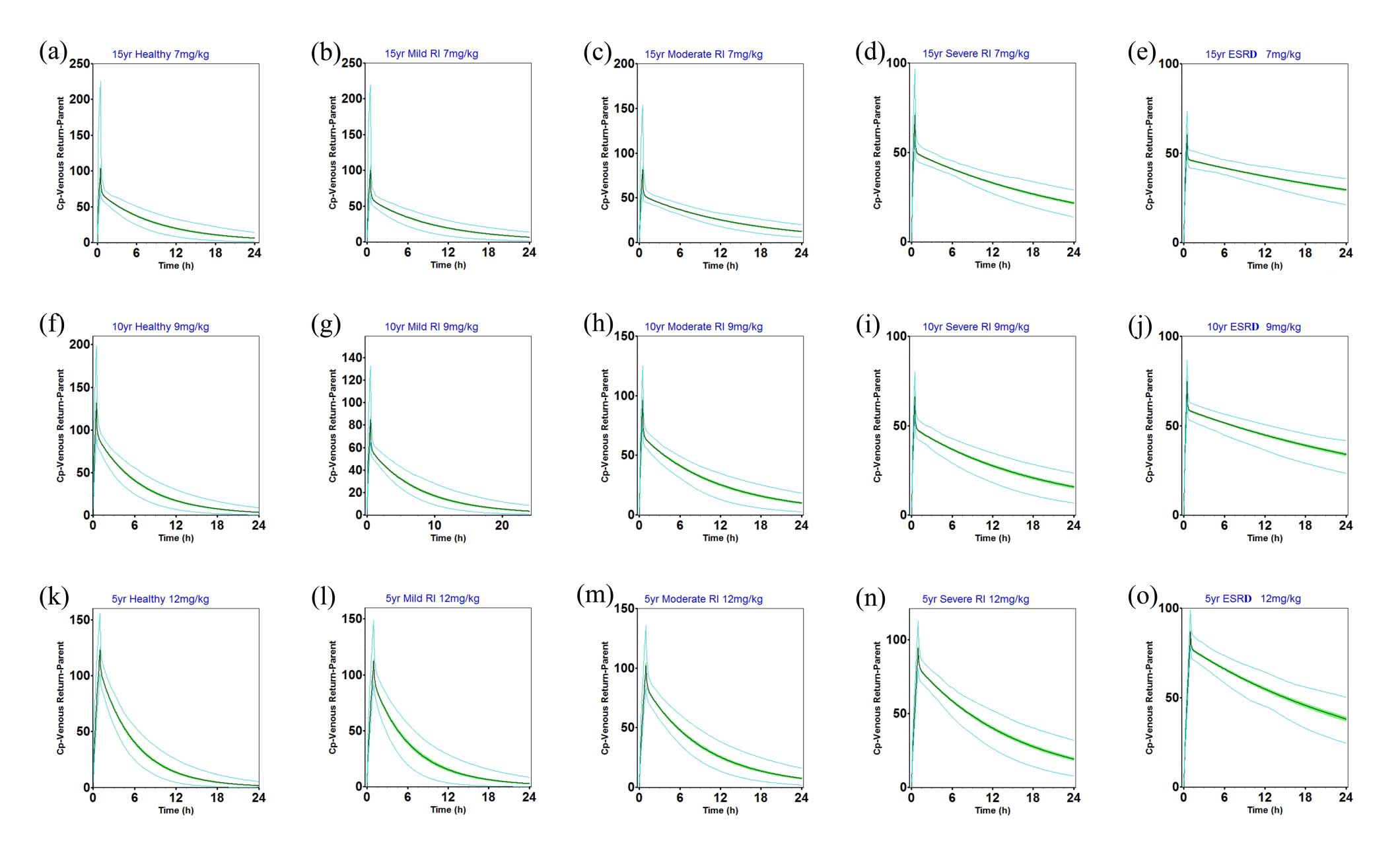
**

**Figure S4.** Population simulation of daptomycinafter administering 7 mg/kg as a single intravenous dose in healthy children aged 12–17 years (**a**) and pediatric patients with various degrees of renal impairment (**b, c, d, e**). 9 mg/kg as a single intravenous dose in healthy children aged 7–11 years (**f**) and pediatric patients with various degrees of renal impairment (**g, h, i, j**). 12 mg/kg as a single intravenous dose in healthy children aged 1–6 years (**k**) and pediatric patients with various degrees of renal impairment (**l, m, n, o**). The shaded area represents the 90% confidence interval for the simulated data, the blue lines indicate the corresponding drug concentration-time curves with a 95% probability. The dosage is from the FDA drug insert for children with *S. aureus* bacteremia.


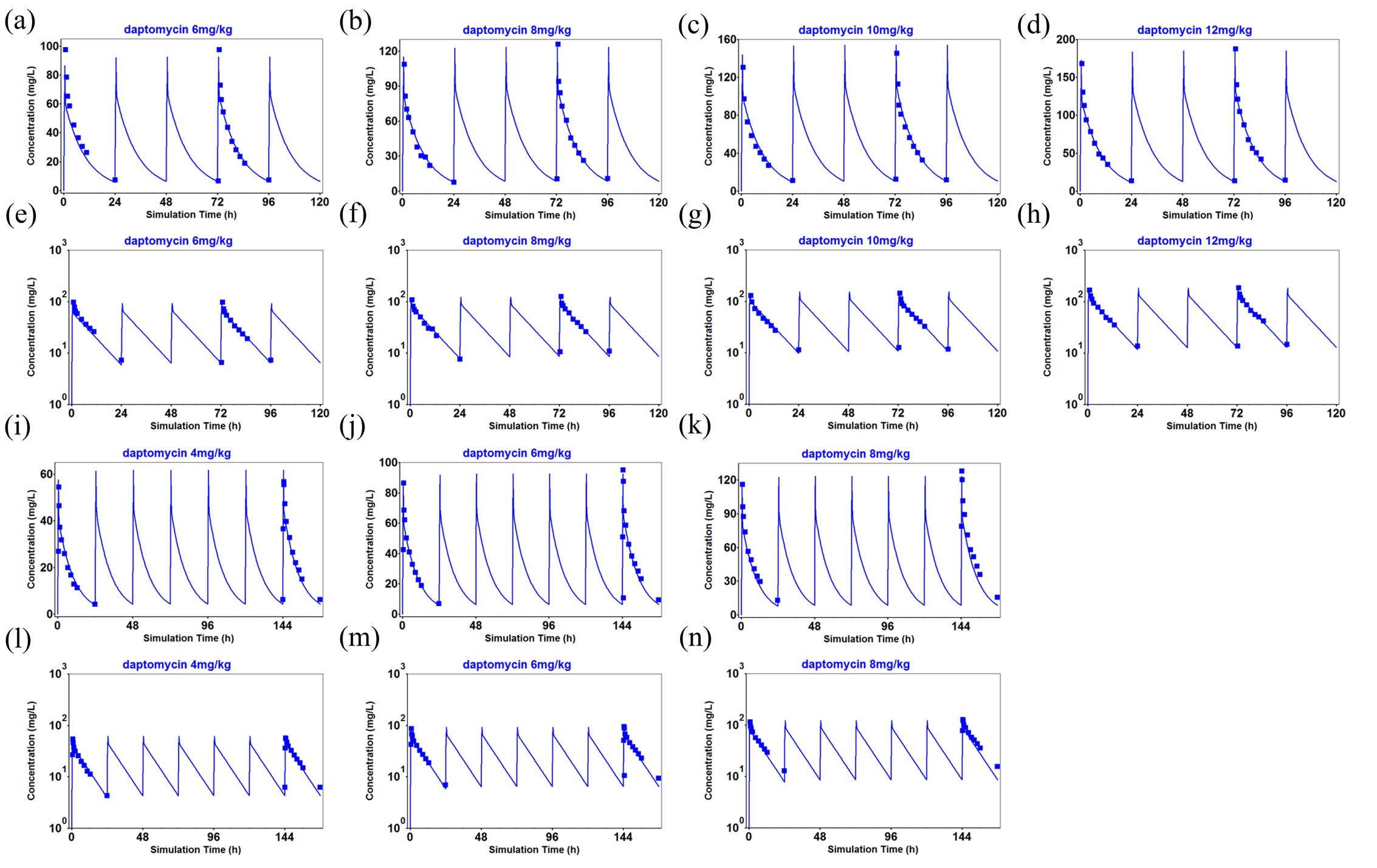


**Figure S5.**Mean drug concentration-time profiles of daptomycin after administering 6 mg/kg (**a, e**), 8 mg/kg (**b, f**), 10 mg/kg (**c, g**) and 12 mg/kg (**d, h**), as multiple intravenous doses for 5 days in healthy adults.4 mg/kg (**i, l**), 6 mg/kg (**j, m**), and 8 mg/kg (**k, n**), as multiple intravenous doses for 7 days in healthy adults. The solid squares represent the observed values. The solid thick lines represent the predicted values.


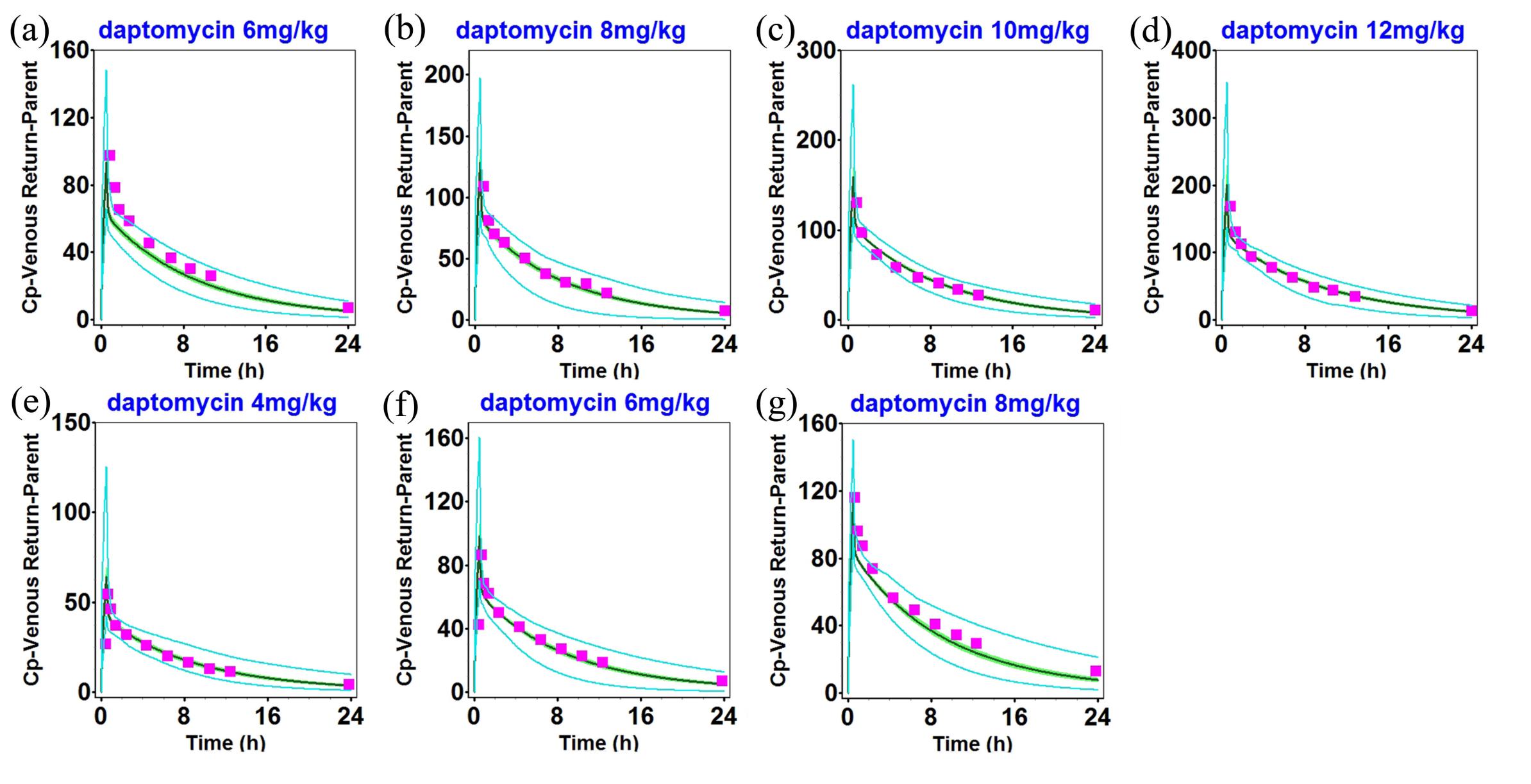


**Figure S6.** Population simulation of daptomycinafter administering 6 mg/kg (**a**), 8 mg/kg (**b**), 10 mg/kg (**c**), 12 mg/kg (**d**) and 4 mg/kg (**e**), 6 mg/kg (**f**), and 8 mg/kg (**g**) as a single intravenous dose in healthy adults. The shaded area represents the 90% confidence interval for the simulated data, the blue lines indicate the corresponding drug concentration-time curves with a 95% probability, and red squares represent the daptomycin concentration derived from the literature.


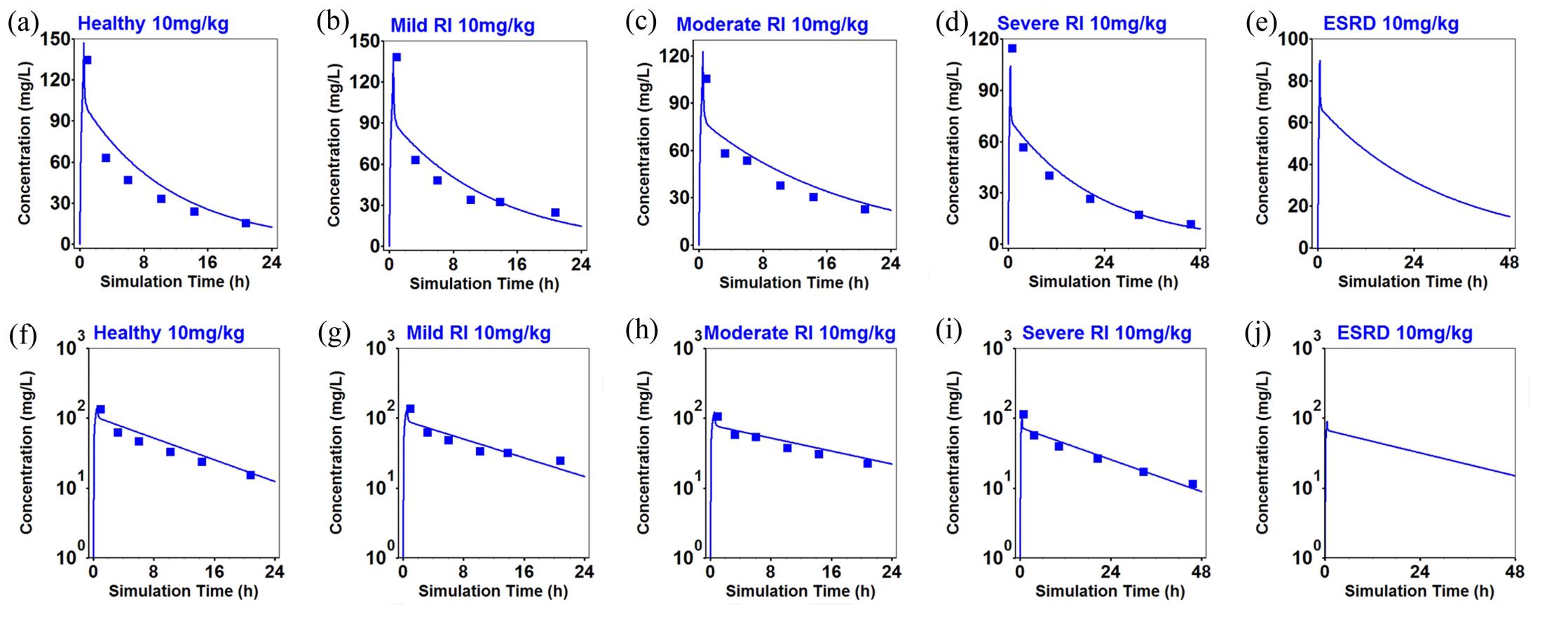


**Figure S7.** Mean drug concentration-time profiles of daptomycin after administering10 mg/kg as a single intravenous dose in healthy adults (**a, f**), adults with mild renal impairment (**b, g**), adults with moderate renal impairment (**c, h**), adults with severe renal impairment (**d, i**), and adults with end-stage renal disease (**e, j**). The solid squares represent the observed values.The solid thick lines represent the predicted values.

**
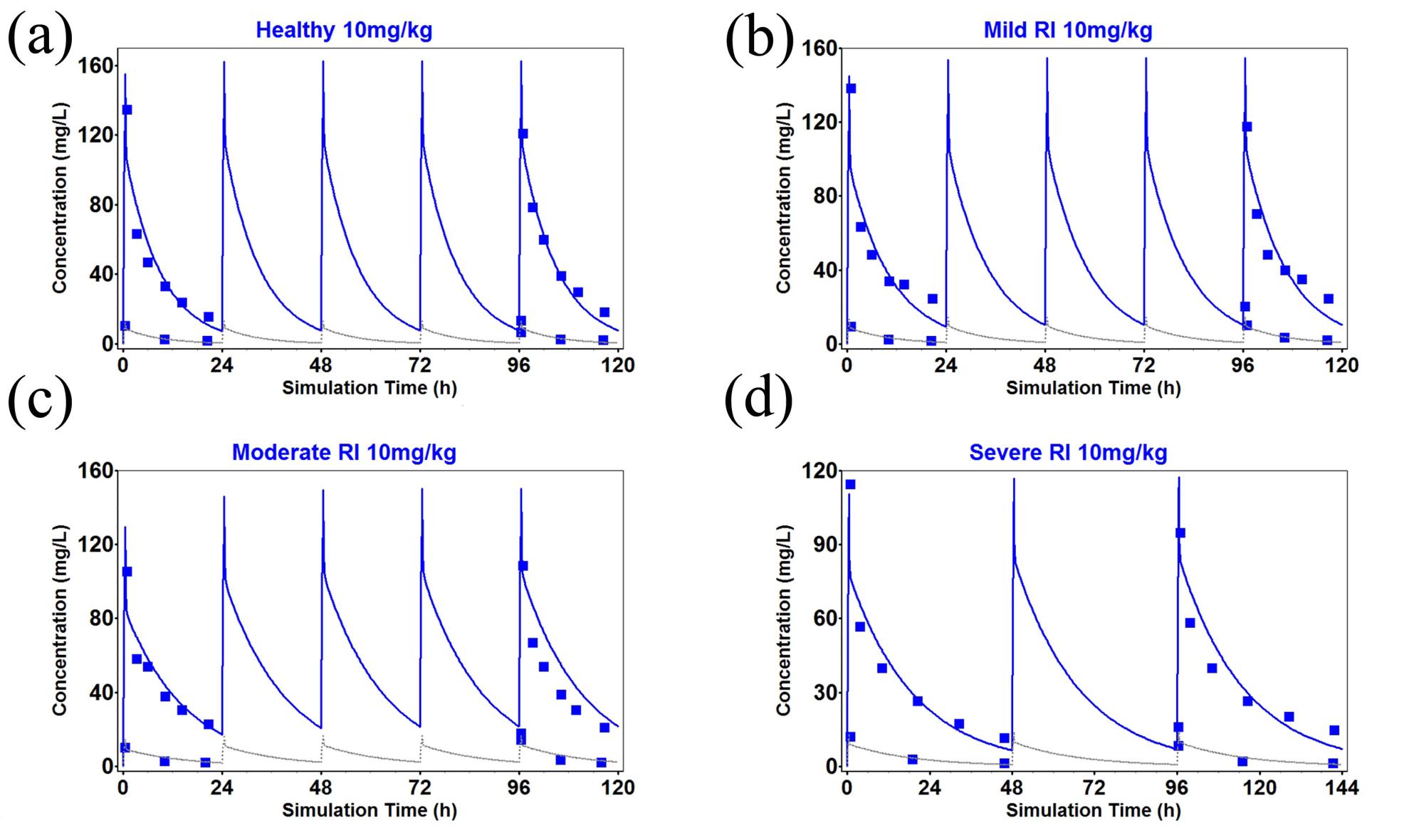
**

**Figure S8.** Mean drug concentration-time profiles of daptomycin after administering10 mg/kg as multiple intravenous doses in healthy adults (**a**), adults with mild renal impairment (**b**), adults with moderate renal impairment (**c**), and adults with severe renal impairment (**d**). Solid squares represent the observed values.Solid thick lines represent the predicted values. The gray dotted lines represent the free drug concentration.


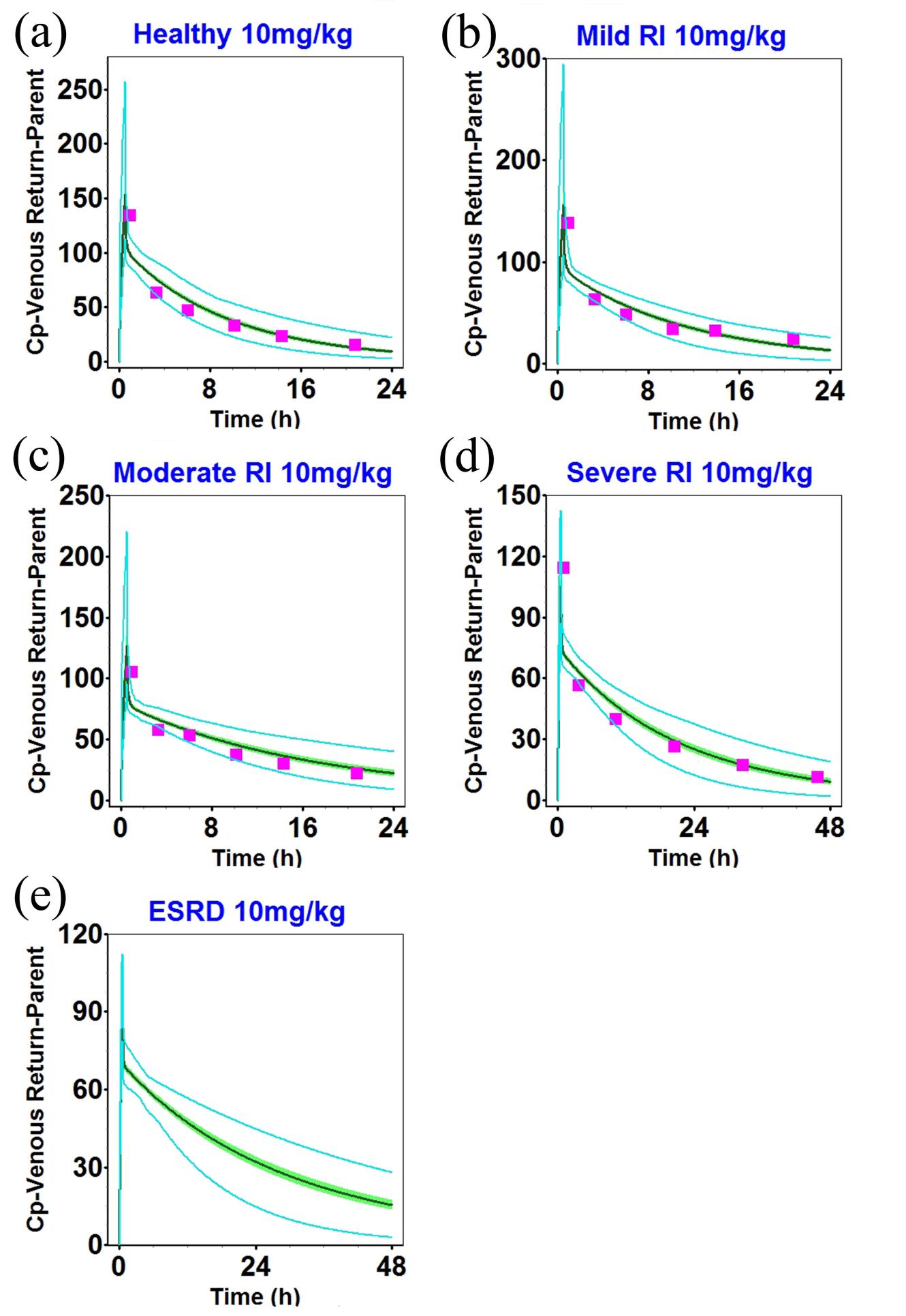


**Figure S9.** Population simulation of daptomycinafter administering 10 mg/kg as a single intravenous dose in healthy adults (**a**), adults with mild renal impairment (**b**), adults with moderate renal impairment (**c**), adults with severe renal impairment (**d**), and adults with end-stage renal disease (**e**). The shaded area represents the 90% confidence interval for the simulated data, the blue lines indicate the corresponding drug concentration-time curves with a 95% probability, and red squares represent the daptomycin concentration derived from the literature.

**Table S1** Average Characteristics of Simulated Population

| **Population** | **Age (yr)** | **Weight (kg)** | **Height (cm)** |
| --- | --- | --- | --- |
| Adults | 40.52 | 71.53 | 166.25 |
| Children | 14.61 | 58.62 | 161.99 |
|  | 8.94 | 34.47 | 136.59 |
|  | 4.02 | 17.61 | 104.17 |
|  | 1.00 | 10.16 | 76.99 |

**Table S2.** Daptomycin Minimum Inhibitory Concentration (MIC) Distributions for *Streptococci*, *Staphylococcus aureus*, and *Enterococcus*

| **Species** | | **No. of isolates inhibited at each concentration (mg/L)** | | | | | | | |
| --- | --- | --- | --- | --- | --- | --- | --- | --- | --- |
|  |  | **0.06** | **0.12** | **0.25** | **0.5** | **1** | **2** | **4** | **8** |
| ***Streptococcus anginosus*** |  | 1 | 5 | 25 | 32 | 3 | 0 | 1 | 0 |
| ***Streptococcus pneumoniae*** |  | 248 | 5121 | 2456 | 280 | 25 | 1 | 0 | 0 |
| ***Staphylococcus aureus* MRSA *^a^*** |  | 0 | 16 | 131 | 244 | 57 | 0 | 0 | 0 |
| ***Staphylococcus aureus* MSSA *^b^*** |  | 0 | 0 | 49 | 338 | 58 | 0 | 0 | 0 |
| ***Enterococcus faecalis*** |  | 9 | 99 | 524 | 3794 | 10066 | 5148 | 525 | 16 |
| ***Enterococcus faecium*** |  | 5 | 39 | 66 | 198 | 988 | 11228 | 3308 | 230 |

***^a^*** MRSA: Methicillin-resistant Staphylococcus aureus

***^b^*** MSSA: [Methicillin-susceptible Staphylococcus aureus](C:/Users/Administrator/AppData/Local/youdao/dict/Application/8.9.3.0/resultui/html/index.html#/javascript:;)

**Table S3.** Minimum Inhibitory Concentration (MIC) Distributions of Daptomycin for *Streptococci*, *Staphylococcus aureus*, and *Enterococcus*

| **Species** | | **Percent of isolates Susceptible at a MIC (mg/L) of** | | | | | | | | **Total No. of Isolates** |
| --- | --- | --- | --- | --- | --- | --- | --- | --- | --- | --- |
|  |  | **0.06** | **0.12** | **0.25** | **0.5** | **1** | **2** | **4** | **8** |  |
| ***Streptococcus anginosus*** |  | 1.49 | 7.46 | 37.31 | 47.76 | 4.48 | 0.00 | 1.49 | 0.00 | 67 |
| ***Streptococcus pneumoniae*** |  | 3.05 | 62.98 | 30.21 | 3.44 | 0.31 | 0.01 | 0.00 | 0.00 | 8131 |
| ***Staphylococcus aureus* MRSA *^a^*** |  | 0.00 | 3.57 | 29.24 | 54.46 | 12.72 | 0.00 | 0.00 | 0.00 | 448 |
| ***Staphylococcus aureus* MSSA *^b^*** |  | 0.00 | 0.00 | 11.01 | 75.96 | 13.03 | 0.00 | 0.00 | 0.00 | 445 |
| ***Enterococcus faecalis*** |  | 0.04 | 0.49 | 2.60 | 18.80 | 49.88 | 25.51 | 2.60 | 0.08 | 20181 |
| ***Enterococcus faecium*** |  | 0.03 | 0.24 | 0.41 | 1.23 | 6.15 | 69.90 | 20.60 | 1.43 | 16062 |

***^a^*** MRSA: Methicillin-resistant Staphylococcus aureus

***^b^*** MSSA: [Methicillin-susceptible Staphylococcus aureus](C:/Users/Administrator/AppData/Local/youdao/dict/Application/8.9.3.0/resultui/html/index.html#/javascript:;)

**Table S4.** Observed and Simulated Pharmacokinetic Parameters of Daptomycin after Intravenous Administration of Different Dosing Regimens in Healthy Children

| **Physiological Status for Model** | | **Age (yr)** | **Dose** | **CL (mL/h/kg)** | | |  | **V_ss_ (mL)** | | |  | **T_half_ (h)** | | |
| --- | --- | --- | --- | --- | --- | --- | --- | --- | --- | --- | --- | --- | --- | --- |
|  | **Age(yr) / Weight(kg) / BMI** |  |  | **Observed** | **Predicted** | **Fold-error** |  | **Observed** | **Predicted** | **Fold-error** |  | **Observed** | **Predicted** | **Fold-error** |
| Healthy | 15 / 70.6 / 22.8 | 12-17 | 4 mg/kg***^a^*** | 11.00 | 9.15 | 1.20 |  | 7766 | 9104 | 1.17 |  | 6.70 | 9.67 | 1.44 |
|  | 10 / 39.7 / 19.1 | 7-11 |  | 17.00 | 13.75 | 1.24 |  | 4764 | 4901 | 1.03 |  | 5.60 | 6.22 | 1.11 |
|  | 5/18/15.68 | 2-6 |  | 20.00 | 20.33 | 1.02 |  | 2340 | 2270 | 1.03 |  | 5.30 | 4.30 | 1.23 |
|  | 15/70.6/22.8 | 12-17 | 5 mg/kg***^b^*** | 11.80 | 9.15 | 1.29 |  | 8200 | 9014 | 1.10 |  | 7.10 | 9.67 | 1.36 |
|  | 10/39.7/19.1 | 7-11 | 7 mg/kg***^b^*** | 13.20 | 13.75 | 1.04 |  | 4470 | 4901 | 1.10 |  | 6.80 | 6.22 | 1.09 |
|  | 5/18/15.68 | 2-6 | 9 mg/kg***^b^*** | 20.80 | 20.33 | 1.02 |  | 2750 | 2270 | 1.21 |  | 4.60 | 4.30 | 1.07 |
|  | 1/10.23/17.1 | 1-2 | 10 mg/kg***^b^*** | 23.10 | 18.57 | 1.24 |  | 1670 | 1226 | 1.36 |  | 4.80 | 4.47 | 1.07 |
|  | 15/70.6/22.8 | 12-17 | 7 mg/kg***^b^*** | 12.40 | 9.15 | 1.36 |  | 6420 | 6088 | 1.05 |  | 7.50 | 6.69 | 1.12 |
|  | 10/39.7/19.1 | 7-11 | 9 mg/kg***^b^*** | 15.90 | 13.75 | 1.16 |  | 4510 | 3661 | 1.23 |  | 6.00 | 4.86 | 1.23 |
|  | 5/18/15.68 | 2-6 | 12 mg/kg***^b^*** | 19.90 | 20.33 | 1.02 |  | 2200 | 1725 | 1.28 |  | 5.10 | 3.29 | 1.55 |

***^a^*** Abdel-Rahman, S.M., Benziger, D.P., Jacobs, R.F., Jafri, H.S., Hong, E.F., and Kearns, G.L. (2008). Single-dose pharmacokinetics of daptomycin in children with suspected or proved gram-positive infections. Pediatr Infect Dis J. 27:330-334. doi: [10.1097/INF.0b013e318160edfc](https://doi.org/10.1097/inf.0b013e318160edfc)

***^b^*** FDA-Label-daptomycin. Available at: <https://www.accessdata.fda.gov/drugsatfda_docs/label/2021/021572s065s066lbl.pdf>
